# Pesticides in surface waters: a comparison with regulatory acceptable concentrations (RACs) determined in the authorization process and consideration for regulation

**DOI:** 10.1186/s12302-016-0083-8

**Published:** 2016-04-22

**Authors:** Katja Knauer

**Affiliations:** Federal Office for Agriculture, Mattenhofstr. 5, 3003 Bern, Switzerland

**Keywords:** Monitoring, Surface water, Regulation, Pesticides, Risk mitigation, Safety

## Abstract

**Background:**

Chemical analysis of surface water conducted in European countries indicates that pesticides are often detected in surface waters. This asks regulatory authorities to consider these monitoring data while re-evaluating pesticide approval and setting appropriate risk mitigation measures. During the years 2005–2012, the cantons in Switzerland performed 345,000 pesticide measurements in surface waters. Overall, 203 approved pesticides were examined. For 60 of these substances, regulatory acceptable concentrations (RACs) were published, which were determined from ecotoxicological data in accordance with international test methods within the framework of the authorization procedure.

**Results:**

For 73 % of the 60 evaluated pesticides, the monitoring data demonstrated that no exceedance of the RAC in surface waters was found. For the 16 remaining compounds, measured environmental concentrations (MECs) were exceeding the RAC value at some sampling sites. However, the 95 percentile of the MECs of all substances analyzed were below the respective RACs. Due to the classification system of surface waters in Switzerland, it became obvious that exceedances of the RAC value occurred in small to medium surface waters. Based on these monitoring data, it can be concluded that mainly herbicides and fungicides were exceeding the RAC; for insecticides only one exceedance was determined. The findings demonstrate that in principle the pesticides are safely used. Most of the exceedances were measured in a surface water surrounded by vineyards in the canton Geneva. Therefore, risk mitigation measures were locally implemented to reduce the entry of pesticides.

**Conclusions:**

Results suggest that a few pesticides in use might account for most of the concern for aquatic life. These pesticides with exceedances of the ecotoxicological thresholds are checked for a possible regulatory action. Implementing further risk mitigation measures might be advisable to reduce the exposure in aquatic systems. This evaluation is an ongoing process. When further RAC values are available, currently Switzerland is re-evaluating authorized pesticides, monitoring data can be evaluated accordingly.

## Background

Agriculture is practiced in Switzerland on an agricultural area of 10,51,063 ha which corresponds to 25 % of Switzerland’s surface [1], in relation to monitoring data from 2005 to 2012. To guarantee a profitable harvest and quality of the agricultural products, fertilizers and plant protection products are used.

It is a declared aim of the agricultural policy in Switzerland to secure a powerful and sustainable agriculture while ensuring no unacceptable impact on the environment [2]. Agricultural areas are intensively used for production, but they are also habitats for a variety of plants and animals [3]. Around 80 % of the Swiss surface waters are small streams and border often directly to agricultural land. They are important habitats for aquatic life [4].

Pesticides are chemical and biological agents that are designed to protect crops against harmful organisms, to regulate the growth of plants or eliminate unwanted plants. Biologically active agents can also have side effects on non-target organisms in the environment in addition to the protective effects obtained for crops. Pesticides can enter edge of field water bodies via spray drift, evaporation and deposition, and after rain events as runoff and erosion or drainage. Surface waters may also receive unwanted amounts of pesticides due to improper cleaning of spray equipment or improper applications in residential areas [5, 6].

During the authorization process, possible entries and resulting concentrations in the edge of field water bodies are predicted on the basis of available experimental fate data and models (EU 1107/2009; e.g., German Exposit model or the European FOCUS model). Switzerland is currently using the German Exposit model to estimate surface water concentrations. The European FOCUS model was recently criticized by Knäbel et al. [7] to fail predicting insecticide concentrations in surface waters. Whether a risk could arise from the use of a pesticide is evaluated prospectively by comparing predicted environmental concentrations (PEC) with threshold concentrations based on ecotoxicological data and assessment factors to account for uncertainties. If unacceptable risk is indicated, restrictions on the use are made [6, 8–10] 1107/2009/EC).

National monitoring programs are used to evaluate the current environmental situation and are conducted for surface, ground and drinking water, soil, and specific indicator species in Europe. They are carried out at regular intervals and as a result an enormous amount of data is available, which are often published in national environmental reports (Swiss [11]; for Europe see activities under Directive 2000/60/EC).

Monitoring data of pesticides in surface waters are very useful for the review of the authorization, even though the routine monitoring is often restricted to larger water bodies and differs therefore from the edge of field approach used in the authorization process. In larger water bodies, concentrations of pesticides are expected to be lower than in the edge of field water bodies. Exceedances of ecotoxicological acceptable concentrations in surface waters indicate a possible need for action to adjust the conditions of use of certain products [12–15].

Chemical analysis of surface waters conducted in European countries and the US indicates that pesticides are often detected in surface waters and that aquatic organisms might be at risk [16–20]. This asks for consideration of these monitoring data while re-evaluating approved pesticides by regulatory authorities and setting appropriate risk mitigation measures. In this project, we compared measured environmental concentrations (MEC) in Swiss surface waters to regulatory acceptable concentrations (RACs) derived from the Swiss registration dossiers to evaluate if approved pesticides are in general used according to the safety instructions or if further regulatory action is needed.

## Results and discussion

### Risk assessment for Swiss surface waters

Measured concentrations of pesticides in Swiss surface waters, including all samples taken also without detection, were evaluated to gain two concentrations, the MEC_max_ (maximum concentration of all measured samples) and the MEC_95 %_ (95 % percentile of all measured samples) for comparison to RAC values (see also [4]). For 60 of these substances, RAC values are published, which were determined from ecotoxicological data which were evaluated within the framework of the Swiss authorization procedure (Table 1, Federal office for agriculture [21]). This approach is more conservative than the one used for the evaluation of a potential risk due to micro-pollutants, which are discharged continuously to surface waters, in Switzerland [22]. The authors used the MEC_90 %_ (90 percentile) and MEC_50 %_ (50 percentile) of the respective active ingredients to determine a possible risk.

**Table 1:** Regulatory acceptable concentrations of pesticides with exceedances of monitoring data in Switzerland. Exceedances (*n*) of regulatory acceptable concentrations (RACs) of pesticides in surface waters (FLOZ) presented as percentage of total measurements and measurements in FLOZ 1–3 surface water bodies and calculated risk quotients [MEC_max_ (maximum measured concentration) in comparison to RACs (regulatory acceptable concentrations)]

Substance			RAC exceedance							FLOZ
Pesticide	H I F	RAC (μg/L)	*n*	Exceedance N (%) (FLOZ 1-3)	N-total (*n*-FLOZ 1-3) (*n* > LOQ)	Events Details: Event (number of exceedances in 1 month)	Events (%)	MEC (µg/L)	RQ = MECmax/RAC	
Aclonifen	H	0.5	1	0.62	161	1	0.62	2.3	4.6	3
				(0.96)	(104)					
					(19)					
Chlortoluron	H	2.4	1	0.02	4841	1	0.02	81.1	34	3
				(0.04)	(2518)					
					(408)					
Isoproturon	H	5.8	6	0.10	6247	2 Details:	0.03		2.0	2
				(0.20)	(2933)	1 (5)		6.2–11.6		3
					(1539)	1		8.4		
Linuron	H	0.7	11	0.23	4684	5 Details:	0.11		25	
				(0.48)	(2314)	2 (8)		0.9–2.9		2
					(561)	1		5.4		2
						1		1.6		3
						1		0.9		1
S-Metolachlor	H	7.0	2	0.02	8966	2 Details:	0.02		17	
				(0.06)	(3813)	1		16.9		2
					(3475)	1		9.8		3
Terbuthylazine	H	1.2	23	0.27	8511	15 Details:	0.18		4.7	
				(0.77)	(2976)	1 (1)		1.6		2
					(1938)	1 (1)		1.7		1
						1 (4)		1.5–1.7		2
						1 (3)		1.3–2.7		2
						1 (2)		1.4–1.6		2
						1 (1)		3.8		2
						1 (3)		1.4–2.8		2
						1 (1)		1.4		2
						1 (1)		2.3		1
						1 (1)		1.4		1
						1 (1)		2.1		5
						1 (1)		1.2		3
						1 (1)		1.7		2
						1 (1)		5.6		3
						1 (1)		1.5		1
Chlorpyrifos-methyl	I	0.1	2	0.07	2967	2 Details:	0.07		1.7	
				(1.04)	(193)	1		0.1		2
					(13)	1		0.17		3
Azoxystrobin	F	3.3	7	0.27	2628	2 Details:	0.08	4.4–11	3.5	
				(0.49)	(1436)	1 (5)		3.4–4.0		2
					(562)	1 (2)				2
Difenconazole	F	0.76	3	0.31	963	2 Details:	0.21	1.1–1.2	1.5	
				(0.44)	(689)	1 (2)		1.0		2
					(171)	1 (1)				1
Epoxyconazole	F	0.43	1	0.08	1329	1	0.08	1.0	2.2	1
				(0.11)	(899)					
					(91)					
Fenpropimorph	F	0.2	2	0.03	5861	2 Details:	0.03		1.4	
				(0.10)	(2021)	1		0.20		3
					(47)	1		0.27		2
Fludioxonil	F	2.3	10	1.52	656	4 Details:	0.61		4.9	
				(2.90)	(345)	1		10		2
					(210)	3 (9)		3–5.5		2
Prochloraz	F	0.55	14	1.7	822	14 Details:	1.7		13	
				(2.12)	(661)	1		0.99		2
					(41)	1		1.1		2
						1		2.7		2
						11		0.6–7.2		1
Spiroxamin	F	0.2	9	1.2	760	9 Details:	1.2		62	
				(1.36)	(661)	1		0.6		1
					(150)	1		12		1
						1		0.25		2
						3		0.25–0.6		1
						1		0.35		2
						1		0.2		2
						1		0.55		2
Tebuconazole	F	1.0	1	0.03	3753	1	0.03	1.7	1.7	1
				(0.10)	(983)					
					(714)					
Trifloxystrobin	F	0.7	4	0.42	963	2 Details:	0.21		4.6	2
				(0.58)	(689)	1 (3)		2.6–3.2		1
					(99)	1		0.8		

Monitoring data were collected from 2005 to 2012 in Switzerland

*H* herbicide, *I* insecticide, *F* fungicide

*n* number of samples with exceedances of the RAC value

*N (%)* percentage of exceedances of total measurements of each pesticide

*N (%) (FLOZ 1–3)* percentage of exceedances of measurements of the pesticide in small and middle surface water bodies (FLOZ 1–3)

*n-total* total measurements of the pesticide

*n-FLOZ 1–3* total measurements of the pesticide in small and middle surface water bodies (FLOZ 1 to 3)

*n* *>* *LOQ* = number of measurements > limit of quantification

*events* = summarizing exceedances of a pesticides measured in one month

*events (%)* = percentage of events of exceedances of total measurements of the pesticide

*risk quotient (RQ)* MECmax/RAC

*FLOZ* order of sections of streams by size (Flussordnungszahl)

Comparing the MEC_95 %_ to the RAC of the evaluated pesticides, no exceedance was determined in any measurement. This means that more than 95 % of the measurements revealed concentrations below the RAC. The risk quotients of the 60 pesticides (RQ = MEC_95 %_/RAC) were lower than 0.415. The data indicate that concentrations in surface waters have been lower by a factor of 2–1000 than the RAC values.

**Fig. 1:**
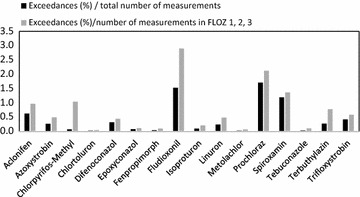
Exceedances of the RAC value as percentage of all measurements and measurements in FLOZ 1–3 per pesticide

Comparing the MEC_max_ with the RAC, it can be concluded that for 44 pesticides the RAC was not exceeded. For the remaining 16 pesticides, however, the RAC has been exceeded (MEC_max_ > RAC) (Table 1). In Fig. 1, the exact percentages of exceedances of the RAC for each pesticide based on all measurements and measurements taken in small to medium surface water bodies (FLOZ 1–3) are shown. Around 120,000 samples were taken in smaller water bodies (FLOZ 1–3) representing edge of the field water bodies and are judged to be suitable for comparison to the RAC obtained under the Swiss pesticide regulation [23]. In Europe, in addition to the pesticide regulation (1107/2009 [24]), the water framework directive (2000/60/EG[25]) aims to protect surface water and defines environmental quality standards (EQS) for larger water bodies. Since both regulations aim to protect different water bodies with different exposure situations, ecotoxicological thresholds also are differently calculated. The typical exposure situation in small water bodies is short-term exposure, whereas in larger systems constant exposure is expected. Wittmer et al. [26] compared therefore measurements in larger surface water bodies in Switzerland to EQS.

Exceedances in Swiss surface waters were mainly determined for herbicides and fungicides; for only one insecticide, chlorpyrifos-methyl, the RAC was exceeded (Fig. 1). For the fungicide prochloraz, the highest number of exceedances was found to be up to 1.7 % of all and 2.1 % of measurements in FLOZ 1–3 (Fig. 1) although the use rates in Switzerland in comparison to the other pesticides with exceedances were low during the years of monitoring. If the extent of exceedances is calculated (RQ = MEC_max_ > RAC), in most cases the RQ was less than 5 (Table 1) indicating a limited risk due to pesticides in smaller water bodies.

Most of the exceedances were determined in a small water body surrounded by vineyards in the canton Geneva. Based on the monitoring results, the canton Geneva implemented locally specific risk mitigation measures to reduce the entry of pesticides and to enhance finally the protection of the surface water [27]. Exceedances might be caused by an enhanced potential for runoff of some herbicides and fungicides (e.g., terbuthylazine, isoproturon, prochloraz, spiroxamin).

This evaluation of the Swiss monitoring data indicates that the risk assessment is in general protective for most situations in the field but might fail in regions with vulnerable conditions such as steep slopes and strong rain events. Herbicides with a potential of runoff were also determined in Swiss surface waters by Moschet et al. [28, 29]. Also in the US, the most frequently detected pesticides in a decadal comparisons were herbicides [20] asking for further risk management for herbicides.

A similar analysis for insecticide on a global scale was performed by Stehle and Schulz [30]. In contrast to our evaluation, the authors compared measurements above the detection limit only to the RAC. They concluded that surface waters globally are at high risk due to insecticides.

**Fig. 2:**
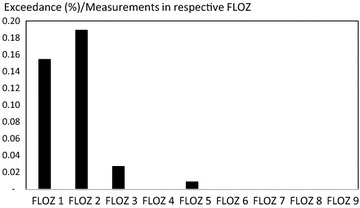
Number of exceedances as percentage of measurements taken in the respective FLOZ in relation to size of the section of the surface water from small (1–2) to medium (3–6) to large (7–9) surface waters

Exceedances of the RAC value occurred mainly in small to medium surface waters in Switzerland (Fig. 2). Similar observation was made by Stehle and Schulz [31] identifying a higher risk for smaller than for larger surface waters.

Exceedances of regulatory acceptable concentrations in surface waters are undesirable. RAC values are derived from the risk assessment as part of the authorization of pesticides and aim to be protective for edge of the field surface water bodies. Analysis of the Swiss monitoring data shows that exceedances of the RAC value were determined when MEC_max_ was used for comparison. Affected were almost exclusively the small and medium sections of running surface waters (FLOZ 1–3). Pesticides with exceedances of RAC value are carefully analyzed to determine possible causes and to define effective risk mitigation measures. Depending on the degree and distribution of the exceedances throughout the country, further risk mitigation might be needed on a local, regional, or national scale.

## Conclusions

Monitoring data are becoming more and more important and part of the re-evaluation procedure of pesticides. However, risk managers have to be aware that the use of monitoring data for this purpose has its limits. The sampling of surface waters is usually done not directly after the application of pesticides, when the highest entries by drift are expected to occur or after rain events, when the highest entries via runoff or drainage are generally occurring [5, 6]. Despite the limitations in the interpretation of monitoring data, a comparison of the measured concentrations in surface waters with the regulatory values derived for plant protection products from the approval process is very useful. Exceedances of these RAC values are indications that the conditions of use of such products, which are defined in the authorization, must be checked. It is therefore necessary to develop suitable post-monitoring schemes and to continue this analysis with other approved pesticides.

## Methods

### Monitoring data from Swiss surface waters

The cantonal water protection offices are responsible for the monitoring of surface waters and carry out regular measurement campaigns. Data from the years 2005 to 2012 were originally compiled by the Federal Office for the Environment and a Switzerland-wide evaluation was published [4]. In total, 345,000 samples were collected mainly from the years 2005 to 2010 and few from the years 2011 to 2012. 120,000 of these samples were collected in small to medium surface water bodies (FLOZ1–3). The sampling strategies were diverse ranging from grap samples to well-designed flow-event triggered sampling. The majority of the measurements were continuously carried out at the International Rhine monitoring station (for further details on sampling strategies see [4]).

### Classification of surface waters by size

In Switzerland, surface waters are classified according to their size and numbers from 1 to 9 and are allocated to the different sections of the streams [Flussordnungszahl (FLOZ)]. For the first section of the stream starting from the spring, the number one is assigned. Where two sections of streams are becoming one bigger section, the ordinal increases by one, if the two sections have the same number, otherwise the higher number is continued [32].

To each river, which was part of the monitoring campaigns, a FLOZ number was assigned. Waters with a FLOZ 1 to 2 are small waters, with a FLOZ 3 to 6 are medium-sized waters, and with a FLOZ 7 to 9 are large water bodies such as the river Rhine.

### Pesticides and the RAC value

During this period of 2005–2012, 203 of the analyzed pesticides were approved for use as PSM ([33], Swiss database on plant protection products). As part of the Swiss approval of pesticides, the regulatory acceptable concentrations (RACs) have been determined for surface water Federal office for agriculture [2, 21]; database on RAC values).

All acute, chronic, and higher tier aquatic studies of the application dossier are considered for that evaluation. Studies are generally carried out under GLP (Good Laboratory Practice) and are in accordance with internationally recognized test methods (OECD). The RAC is based on the test result of the most sensitive species, which is provided with a safety factor to take into account unavoidable uncertainty in the transfer of results obtained from laboratory studies with individual organisms or semi-field experiments to natural water systems [8]. If the RAC value is based on the results of a field studies, e.g. mesocosm studies, transient effects on algae and invertebrates might be accepted. This value corresponds thus to the concentration which should guarantee that no unacceptable effects on aquatic organisms will occur after short- and long-term exposure in surface waters. Since 2012, RAC values are published on the Homepage of the Federal office for agriculture. So far RAC values for 100 substances are published. Currently, Switzerland is re-evaluating authorized products and further RAC values will be published in the coming years. For 60 of these pesticides, monitoring data were available and evaluated in this comparison.

### Evaluating environmental risk with monitoring data

For the entire dataset including also samples below the limit of detection, the maximum concentration (MEC_max_) and the 95 % percentile of all measurements for each pesticide (MEC_95 %_) were determined.

To evaluate the risk for aquatic organisms due to pesticide exposure, the MECs of surface waters were compared to regulatory acceptable concentration (RAC) which were derived from the registration data for plant protection products (risk quotient RQ = MEC/RAC) (Table 1). If the RAC values are not exceeded in surface water, it can be assumed that no unacceptable effect on aquatic species will occur. If the RAC value is lower than the MEC, a risk for aquatic organisms cannot be excluded. Since often measurements were taken in weekly intervals, measurements obtained in a month were grouped together as an event of exceedance to enhance comparability of the data for each pesticide. A comparison of the RAC values with MECs is a potential verification of the correct application of plant protection products or the need for further mitigation (Table 1).

## Abbreviations

MEC: measured environmental concentration
PEC: predicted environmental concentration
RAC: regulatory acceptable concentration
RQ: risk quotient

## References

[1] Swiss Federal Statistical Office (2012). Data base on land use. http://www.bfs.admin.ch/bfs/portal/de/index/themen/07/03/blank/data/01/02.html. Accessed May 2015

[2] Federal office for agriculture (2015) Swiss agricultural report. Available via DIALOG. http://www.agrarbericht.ch/de. Accessed 30 May 2015

[3] Davies B, Biggsa J, Williams P, Whitfield M, Nicolet P, Sear D, Bray S, Maund S (2008). Comparative biodiversity of aquatic habitats in the European agricultural landscape. Agri Ecosyst Environ.

[4] Munz I, Leu C, Wittmer I (2012). Schweizweite Auswertung von Pestizidmessungen in Fliessgewässern. Aqua Gas.

[5] Wauchope RD (1978). The pesticide content of surface water draining from agricultural fields—a review. J Environ Qual.

[6] Schulz R (2004). Field studies on exposure, effects, and risk mitigation of aquatic nonpoint-source insecticide pollution: a review. J Environ Qual.

[7] Knäbel A, Sthele S, Schäfer R, Schulz R (2012). Regulatory FOCUS surface water models fail to predict insecticide concentrations in the field. Environ Sci Technol.

[8] European Food and Safety Agency (2013). Scientific opinion—guidance on tiered risk assessment for plant protection products for aquatic organisms in edge-of-field surface waters; European Food Safety Authority (EFSA), EFSA Panel on Plant Protection Products and their Residues (PPR), Parma, Italy. EFSA Journal.

[9] Reichenberger S, Bach M, Skitschak A, Frede HG (2007). Mitigation strategies to reduce pesticide inputs into ground- and surface water and their effectiveness; a review. Sci Tot Environ.

[10] Alix A, Knauer K, Streloke M, Poulsen V (2015) Development of a harmonized risk mitigation toolbox dedicated to environmental risks of pesticides in farmland in Europe: outcome of the MAgPIE workshop. JKI Spise. (in press)

[11] Federal office for the environment (2016) http://www.bafu.admin.ch/wasser/13465/13483/14090/14128/index.html?lang=en. Accessed March 2016

[12] Crommentuijn T, Sijm D, de Bruijn J, van Leeuwen K, van de Plassche E (2000). Maximum permissible and negligible concentrations for some organic substances and pesticides. J Environ Manag.

[13] Kreuger J, Nilsson E (2001). Catchment scale risk-mitigation experiences—key issues for reducing pesticide transport to surface waters. BCPC Symp Proc.

[14] Boye K, Jarvis N, Moeys J, Gönczi M, Kreuger J (2012). Pesticide run-off to Swedish surface waters and appropriate mitigation strategies: a review of the knowledge focusing on vegetated buffer strips.

[15] Bundschuh M, Goedkoop W, Kreuger J (2014). Evaluation of pesticide monitoring strategies in agricultural streams based on the toxic-unit concept—experiences from long-term measurements. Sci Tot Environ.

[16] Müller K, Bach M, Hartmann H, Spiteller M, Frede H (2002). Point- and nonpoint-source pesticide contamination in the Zwester Ohm catchment, Germany. J Environ Qual.

[17] Irace-Guigand S, Aaron JJ, Scribe P, Barcelo D (2004). A comparison of the environmental impact of pesticide multiresidues and their occurrence in river waters surveyed by liquid chromatography coupled in tandem with UV diode array detection and mass spectrometry. Chemosphere.

[18] Chèvre N, Loepfe C, Singer H, Stamm C, Fenner K, Escher BI (2006). Including mixtures in the determination of water quality criteria for herbicides in surface water. Environ Sci Technol.

[19] Malaj E, von der Ohe PC, Grote M, Kuhne R, Mondy CP, Usseglio-Polatera P, Brack W, Schafer RB (2014). Organic chemicals jeopardize the health of freshwater ecosystems on the continental scale. Proc Natl Acad Sci USA.

[20] Stone WW, Gilliom RJ, Ryberg KR (2014). Pesticides in U.S. Streams and Rivers: occurrence and Trends during 1992–2011; 2014. Environ Sci Technol.

[21] Federal office for agriculture (2014) Data base on regulatory acceptable concentrations of plant protection products. http://www.blw.admin.ch/themen/00011/00075/00224/index.html?lang=de. Accessed 30 May 2014

[22] Götz C, Kase R, Hollender J (2010). Mikroverunreinigungen—Beurteilungskonzept für organische Spurenstoffe aus kommunalem Abwasser.

[23] Swiss pesticide regulation (2015) Verordnung über das Inverkehrbringen von Pflanzenschutzmitteln (Pflanzenschutzmittelverordnung, PSMV) 916.161; 12. Mai 2010 (Stand am 17. November 2015)

[24] European Commission (2011). Implementing Regulation 1107/2009/EC of the European parliament and of the Council as regards uniform principles for evaluation and authorization of plant protection products. Off J Euro.

[25] European Commission (2000) Directive 2000/60/EC of the European parliament and of the council of 23 October 2000 establishing a framework for community action in the field of water policy. Off J Eur L327

[26] Wittmer I, Moschet C, Simovic J, Singer H, Stamm C, Hollender J, Junghans M (2014). Über 100 Pestizide in Fliessgewässern. Aqua Gas.

[27] Canton Geneva (2015) The implementation of risk mitigation measures in the agricultural landscape to reduce pesticide entry to surface water Ruisseau des Charmilles, Switzerland. Available via DIALOG. http://ge.ch/agriculture/informations-professionnelles/protection-des-plantes/residus-phytosanitaires. Accessed 30 March 2016

[28] Moschet C, Wittmer I, Simovic J, Junghans M, Piazzoli A, Singer H, Hollender J (2014). How a complete pesticide screening changes the assessment of surface water quality. Environ Sci Technol.

[29] Moschet C, Vermeirssen ELM, Seiz R, Pfefferli H, Hollender J (2014). Picogram per liter detections of pyrethroids and organophosphates in surface waters using passive sampling. Wat Res.

[30] Stehle S, Schulz R (2015). Agricultural insecticides threaten surface waters at the global scale. Proc Natl Acad Sci USA.

[31] Stehle S, Schulz R (2015). Pesticide authorization in the EU—environmental unprotected?. Environ Sci Pollut Res.

[32] Federal office for the environment (2013) Classification of surface waters by size. Available via DIALOG. http://www.bafu.admin.ch/hydrologie/01835/02118/02120/index. html? lang = en

[33] Federal office for agriculture (2016) Bern, Switzerland. Data base on authorized plant protection products. http://www.blw.admin.ch/psm/produkte/index.html?lang=de. Accessed 30 May 2015

